# Network pharmacology and molecular docking technology for exploring the effect and mechanism of Radix Bupleuri and Radix Paeoniae Alba herb-pair on anti-hepatitis: A review

**DOI:** 10.1097/MD.0000000000035443

**Published:** 2023-12-01

**Authors:** Long Huang, Qingsheng Yu, Hui Peng, Zhou Zhen

**Affiliations:** a Department of No. 1 Surgery, The first hospital affiliated to Anhui University of Traditional Chinese Medicine, Hefei, Anhui Province, China; b Department of Surgery, The Second Hospital Affiliated to Anhui University of Traditional Chinese Medicine, Hefei, Anhui Province, China.

**Keywords:** hepatitis, molecular docking, network pharmacology, Radix Bupleuri, Radix Paeoniae Alba

## Abstract

The Radix Bupleuri and Radix Paeoniae Alba herb-pair (RRH) are the most classic compatible drug pair for the treatment of hepatitis. However, the underlying mechanism remains unclear. Therefore, network pharmacology and molecular docking were conducted to investigate the prospective therapeutic constituents, targets, and pharmacological mechanisms of RRH in the treatment of hepatitis. The active components of RRH from the TCMSP database and disease-related targets from the OMIM, PharmGkb, GeneCards, TTD, and DrugBank databases were identified. The “drug-target-disease” network diagram and protein–protein interaction (PPI) network were constructed using Cytoscape (v3.8.0) and Online STRING 11.0. GO and KEGG pathway enrichment analyses were performed using R version 4.1.2, and molecular docking was performed to verify the results. We placed 176 overlapping cross genes into Online STRING 11.0 and obtained 14 core targets. A “Component-Target-GO-KEGG” network diagram was constructed, which was composed of 7 components, 14 targets, 10 biological processes, and 10 signal pathways. A total of 2413 GO biological processes and 174 KEGG pathways were explored for hepatitis treatment. Quercetin, kaempferol, isorhamnetin, and beta-sitosterol, which are the main bioactive components, were employed to bind the disease's hub targets, ensuring fulfillment of spatial and energy matching. The anti-hepatitis mechanism of RRH may be associated with several targets including RELA, AKT1, JUN, MAPK1, TP53, CCND1, MYC, NFKBIA, CDKN1A, and their respective signaling pathways. The main bioactive components in RRH, including quercetin, kaempferol, isorhamnetin, and beta-sitosterol, were used to bind the hub targets of the disease, which may provide insights into drug development for hepatitis.

## 1. Introduction

Hepatitis is an important public health problem worldwide and its harm to the world is obvious. According to the statistics of the World Health Organization, the number of chronic hepatitis B patients worldwide is as high as 250 million, accounting for approximately 3.5% of the world total population.^[1]^ China is the world largest burden of HBV infection, about 7 million people develop advanced liver disease or liver cancer every year, of which 0.38 million people die of liver cancer.^[2]^ Given the seriousness of the epidemic of hepatitis virus infection, despite the continuous development of new drugs and the continuous improvement of antiviral drugs in recent years, the complete cure of chronic hepatitis remains a clinical problem and other more effective drug treatments need to be found. There are various causes of hepatitis, including hepatitis virus infection, alcoholic hepatitis, nonalcoholic fatty liver disease, autoimmune hepatitis, and chemical drugs. Despite the availability of diverse antiviral and liver-protective drugs, the majority of them are designed to target only one specific therapeutic site. As a result, the development of effective liver-protective drugs with multiple components, targets, and pathways remains insufficient. Drug treatments for various types of hepatitis are still in the exploration stage. Therefore, it is of great significance for human health care to develop new drugs and explore new therapeutic targets for hepatitis treatment. This article aims to identify effective components and specific targets for hepatitis treatment using network pharmacology in RRH. Additionally, it aims to establish a theoretical basis for the development of multi-component, multi-target, and multi-path liver protection drugs through molecular docking validation.

Previous studies have investigated the mechanisms of peach kernel and safflower in the prevention of liver fibrosis, utilizing network pharmacology and molecular docking methods.^[3]^ Since liver fibrosis is a manifestation of hepatitis, the primary objective of this study is to further investigate the important constituents of traditional Chinese medicine that possess anti-hepatitis properties, through in silico modeling validation. Radix Bupleuri and Radix Paeoniae Alba herb-pair (RRH) are the most classic compatible drug pairs for the treatment of chronic liver disease.^[4,5]^ It is also the core drug of many classic and famous prescriptions for the treatment of liver disease, such as Xiaoyao Powder, Chaihu-Shugan-San, and Sini Powder, which have many functions, such as restoring liver function and inhibiting liver fibrosis.^[6–8]^ In these formulas, Radix Bupleuri plays a major role, and Radix Paeoniae Alba plays an auxiliary role. However, the mechanism of RRH in the anti-hepatitis effect of RRH remains unclear. Therefore, it is important to clarify the effective components of RRH for the treatment of hepatitis and explore new therapeutic targets using network pharmacology and molecular docking technology.

Network pharmacology systematically predicts the possible mechanism of drug treatment of diseases by building a “component-gene target-disease” network diagram based on gene ontology (GO) and Kyoto encyclopedia of genes and genomes (KEGG) enrichment analyses.^[9]^ Furthermore, the binding mode and intensity of drug ligands and targets can be predicted using molecular docking technology.^[10]^ In this study, we conducted a comprehensive network pharmacology study based on network pharmacology and molecular docking technology to explore the possible pharmacological mechanism of RRH in the treatment of hepatitis, including screening of key ingredients and exploring new therapeutic targets, to lay a theoretical foundation for the development of new drugs for hepatitis treatment.

## 2. Methods

The traditional Chinese medicine systems pharmacology database and analysis platform (TCMSP) (http://tcmspw.com/tcmsp.php) was used to screen for key ingredients and targets of RRH. Five databases, including GeneCards Human Gene database (GeneCards, https://www.genecards.org/), online mendelian inheritance in man database (OMIM, https://omim.org/), therapeutic target database (TTD, https://db.idrblab.net/ttd/), DrugBank Database (https://go.drugbank.com/), and pharmacogenetics and pharmacogenomics knowledge base (pharmGKB, https://www.pharmgkb.org/), were used to search for disease-related targets. We used Cytoscape 3.8.0, STRING Databases 11.5, AutoDockTools version 1.5.6 for network construction, protein-protein interaction (PPI) information collection, and molecular docking verification. R version 4.1.2 was used to explore the mechanism of RRH in treating hepatitis based on GO and KEGG enrichment analysis.

### 2.1. Screening of compound components from RRH

The active components and drug targets of RRH were explored using TCMSP. By screening the drug components of Radix Bupleuri and Radix Paeoniae Alba, we defined drug molecules with oral bioavailability ≥ 30% and drug similarity ≥ 0.18 as active molecules. The corresponding target gene information was searched for and annotated using the UniProt database (https://www.uniprot.org/).^[11]^ The active components and related targets in RRH were screened from TCMSP,^[12]^ and the targets were annotated using the UniPort and GeneCards databases.^[13]^

### 2.2. Acquisition of disease targets

The keyword “Hepatitis” was used to screen disease-related targets from 5 databases, including GeneCards, OMIM, TTD, DrugBank Database, and pharmGKB. In the next step, we merged all collected disease-related targets and removed duplicate targets. Finally, overlapping intersection genes between the active component targets of RRH and the target genes in hepatitis were obtained.

### 2.3. Construction of the component-target-GO-KEGG network diagram

The components and disease-related targets of RRH in the treatment of hepatitis were imported into Cytoscape 3.8.0, and then a “component-target-disease” network was constructed and visualized.^[14]^ We constructed a network diagram of the hub nodes according to the hub nodes. To investigate the relationship between components, core targets, biological processes (BP), and signaling pathways, we built a “Components-Core Targets-GO-KEGG” network diagram concurrently. Target molecules, including components, BPs, signaling pathways, and disease-related target genes, are represented as nodes. The interactions between the nodes are represented as edges.

### 2.4. PPI network of target protein interaction

The overlapping intersection genes between the active component targets of RRH and target genes in hepatitis were imported into Online STRING 11.0 to construct a visualized PPI network diagram. The confidence score in PPI was set to 0.9 for the highest confidence from the STRING database.^[15]^ The amplified target proteins of RRH in the STRING database were input into Cytoscape software V3.8.0, and then the 6 parameters of “Betweenness,” “Closeness,” “Degree,” “Eigenvector,” “Local Average Connectivity-based method,” and “Network” were obtained by CytoNCA tool. The median values of these parameters were calculated, and all nodes whose 6 parameters were greater than the median values were selected as hub nodes.^[16]^

We repeated the screening of these 6 parameters to obtain the core target of the disease. Cytoscape was used to construct a visual network map between core targets, key ingredients, BPs, and signal pathways.

#### 2.4.1. Gene ontology and pathway enrichment analyses.

R version 4.1.2 was used for GO functional annotation and KEGG pathway analysis of RRH in the treatment of hepatitis, and the enrichment analysis results were visualized.^[17]^ The GO database, including BP, molecular function, and cellular component, was used to explore the potential biological molecular mechanisms.^[18]^ The KEGG database has also been used to identify biological functions and candidate targets.^[19]^

### 2.5. Molecular docking verification

The structures of the RRH components and key targets were downloaded from the PubChem database (https://pubchem.ncbi.nlm.nih.gov/) and the protein database database (http://www.rcsb.org/pdb/home/home.do), respectively. The molecular docking protocol was performed by comparing the binding positions and binding energies of each ligand. The planar structure of the 4 ligands, namely kaempferol, quercetin, beta-sitosterol, and isorhamnetin, was converted into a 3-dimensional structure using ChemBio3D software (Version 14.0.0.117). Subsequently, the MM2 function optimized the force field of each ligand and saved the processed ligands in *.mol2 format. Finally, AutoDockTools Version 1.5.6 converted each ligand to *.pdbqt format. After removing water molecules and original ligand molecules, as well as undergoing hydrogenation using PyMOL Version 2.4.0, the key target proteins were converted to *.pdbqt format using AutoDockTools Version 1.5.6. The Ligand Docking module was used to verify the reliability of the results using AutoDockTools Version 1.5.6, and the bonding activity of the component to the key targets was evaluated using the binding energy.^[20]^ The grid size (60 Å × 60 Å × 60 Å) was set for ligand molecules and receptor molecules, with the center of the grid aligned with the ligand. The molecular docking employed a semi-flexible docking method, allowing for ligand conformational changes. The Lamarckian genetic algorithm was utilized with the number of docking rounds set to 100, while all other parameters were maintained at their default values. Subsequently, the Autodock docking program was executed for molecular docking. The binding energy was used as a reference indicator to obtain the docking values between each ligand and receptor protein. The optimal docking result with the lowest binding energy was then screened out for further analysis.

## 3. Results

### 3.1. Components and targets of RRH and Hepatitis related targets from 5 databases

Based on the TCMSP database, 17 components of Radix Bupleuri and 13 components of Radix Paeoniae Alba met the criteria of oral bioavailability ≥ 30% and drug similarity ≥ 0.18. Based on the pharmacology database and analysis platform of TCMSP, there were 181 potential drug component targets in RRH, including 171 in Radix Bupleuri and 77 in Radix Paeoniae Alba.

**Figure 1.**
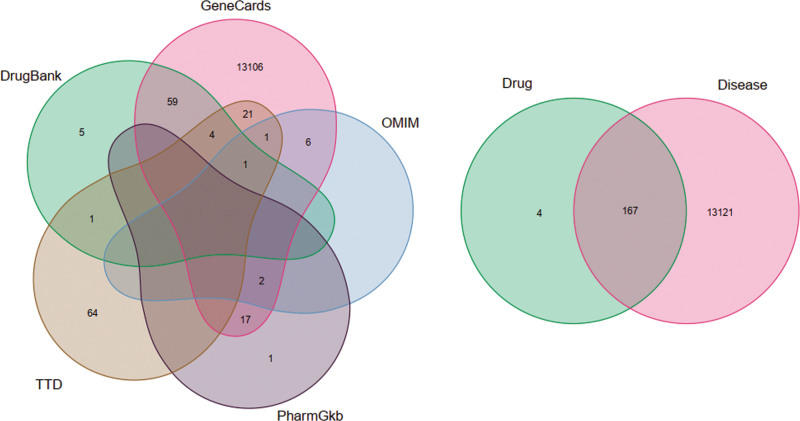
Overlapped genes between targets of active components in Radix Bupleuri and Radix Paeoniae Alba herb-pair and hepatitis related genes from the GeneCards, DrugBank, OMIM, TTD, and pharmGKB databases. (A) Hepatitis related genes; (B) overlapped genes. OMIM = online mendelian inheritance in man database, pharmGKB = pharmacogenetics and pharmacogenomics knowledge base, TTD = therapeutic target database.

In addition, the disease-related gene targets were screened from the GeneCards, DrugBank, OMIM, TTD, and pharmGKB databases, and finally, we obtained 13288 disease-related targets. By comparing the intersection of drug targets in RRH and disease-related targets, 176 intersection genes overlapped between 181 target proteins of the active components and 13288 potential disease targets (Fig. 1).

### 3.2. Network diagram

**Figure 2.**
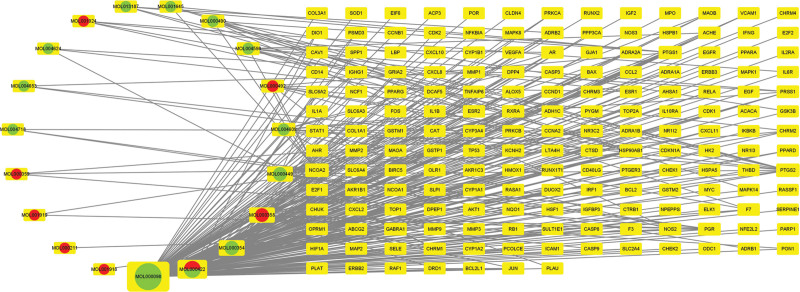
The construction of the Component-Target-Disease network diagram.

For the treatment of hepatitis, a “component-target-disease” network diagram was constructed, which was composed of 195 nodes and 328 edges. The network diagram represents the relationships between the 19 RRH components and 176 disease-related gene targets. The network results of RRH for hepatitis treatment are shown in Figure 2.

### 3.3. PPI network

**Figure 3.**
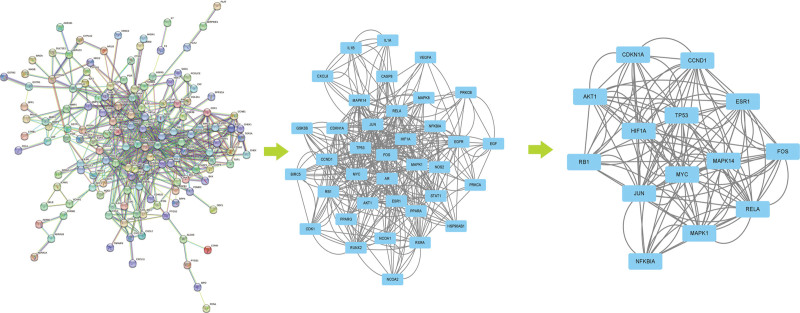
Map of protein interaction network and the identification of hub genes.

We placed 176 overlapping cross genes between the active component targets of RRH and target genes in hepatitis into Online STRING 11.0 (https://string-db.org/) to build a visual PPI network diagram, with a confidence score of 0.9. The PPI network diagram was composed of 175 nodes and 556 edges (Fig. 3).

**Figure 4.**
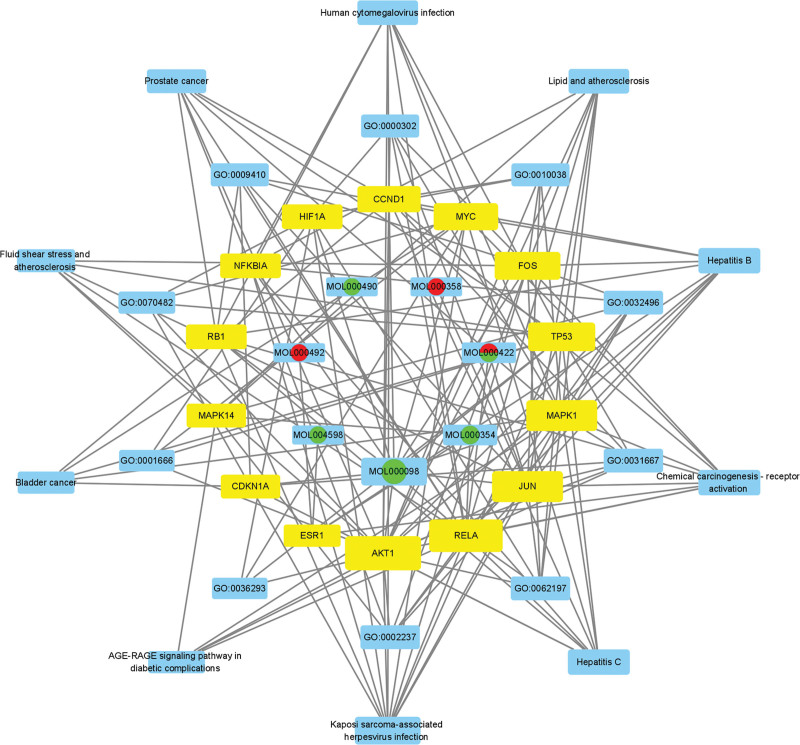
The construction of the Component-Core Target-GO-KEGG network diagram. GO = gene ontology, KEGG = Kyoto encyclopedia of genes and genomes.

Furthermore, we use the CytoNCA tool in Cytoscape to obtain the hub targets of RRH, based on the following 6 parameters of “Degree,” “Betweenness,” “Closeness,” “Eigenvector,” “Local Average Connectivity,” and “Network.” The final network diagram was constructed by selecting targets corresponding to parameters above the median value. The network diagram was composed of 14 hub nodes and 132 edges (Fig. 4).

To investigate the relationship between components, core targets, BPs, and signal passways, we built a “Component-Core Target-GO-KEGG” network diagram, which was composed of 7 components, 14 targets, 10 BPs, and 10 signal pathways. The main components and core targets of the degree in RRH are listed in Tables 1 and 2, respectively.

**Table 1:** The key effective components of Radix Bupleuri and Radix Paeoniae Alba herb-pair.

MolId	Molecule name	Herb	Structure	OB (%)	DL	Degree
MOL000098	Quercetin	Chaihu	C15H10O7	46.43	0.28	134
MOL000422	Kaempferol	Chaihu/Baishao	C15H10O6	41.88	0.24	51
MOL000354	Isorhamnetin	Chaihu	C16H12O7	49.6	0.31	28
MOL000358	Beta-sitosterol	Baishao	C29H50O	36.91	0.75	26
MOL000492	(+)-Catechin	Baishao	C15H14O6	54.83	0.24	8
MOL004598	3,5,6,7-tetramethoxy-2-(3,4,5-trimethoxyphenyl) chromone	Chaihu	C22H24O9	31.97	0.59	8
MOL000490	Petunidin	Chaihu	C16H13O7+	30.05	0.31	8

DL = drug similarity, OB = oral bioavailability.

**Table 2:** Core targets of hepatitis.

Name	Description	UniProt	Length	Degree	PDB DOI
RELA	Transcription factor p65	Q04206	551	3	10.2210/pdb2VGE/pdb
AKT1	RAC-alpha serine/threonine-protein kinase	P31749	480	2	10.2210/pdb1H10/pdb
JUN	Transcription factor AP-1	P05412	331	3	10.2210/pdb1A02/pdb
MAPK1	Mitogen-activated protein kinase 1	P28482	360	1	10.2210/pdb2DKZ/pdb
TP53	Cellular tumor antigen p53	P04637	393	1	10.2210/pdb1GZH/pdb
CCND1	G1/S-specific cyclin-D1	P24385	295	1	10.2210/pdb2W99/pdb
MYC	Myc proto-oncogene protein	P01106	439	1	10.2210/pdb1EE4/pdb
ESR1	Estrogen receptor (ER)	P03372	595	3	10.2210/pdb1A52/pdb
RB1	Retinoblastoma-associated protein	P06400	928	1	10.2210/pdb8DWK/pdb
MAPK14	Mitogen-activated protein kinase 14	Q96GX9	242	2	10.2210/pdb1KWP/pdb
NFKBIA	NF-kappa-B inhibitor alpha	P25963	317	1	10.2210/pdb1IKN/pdb
FOS	Proto-oncogene c-Fos	P01100	380	1	10.2210/pdb1FOS/pdb
CDKN1A	Cyclin-dependent kinase inhibitor 1	P38936	164	1	10.2210/pdb7KYQ/pdb
HIF1A	Hypoxia-inducible factor 1-alpha	Q16665	826	1	10.2210/pdb1H2K/pdb

### 3.4. GO and KEGG pathway enrichment analyses

**Figure 5.**
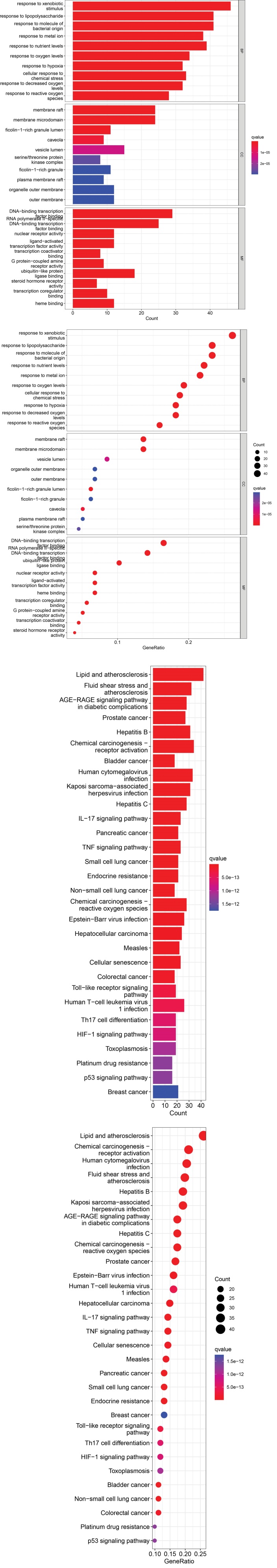
Gene ontology (GO) and Kyoto encyclopedia of genes and genomes (KEGG) enrichment analysis of the target proteins. (A) GO bar chart; (B) GO bubble chart; (C) KEGG bar chart; (D) KEGG bubble chart.

A total of 2413 GO BPs and 174 KEGG pathways were obtained after the enrichment analysis of 176 overlapping cross genes (*P* < .01). To further investigate 2413 GO BPs, 2142 BPs, 61 cellular component terms, and 209 molecular functions were found to be enriched in GO enrichment analysis (*P* < .01). The top 10 GO BPs are listed as follows: (GO:0009410) response to xenobiotic stimulus, (GO:0032496) response to lipopolysaccharide, (GO:0002237) response to molecule of bacterial origin, (GO:0010038) response to metal ion, (GO:0031667)response to nutrient levels, (GO:0070482) response to oxygen levels, (GO:0001666) response to hypoxia, (GO:0062197) cellular response to chemical stress, (GO:0036293) response to decreased oxygen levels, and (GO:0000302) response to reactive oxygen species (Fig. 5A and B).

The first 30 KEGG pathways were obtained for analysis based on *P* value. The top 10 KEGG pathways are as follows: Lipid and atherosclerosis signaling pathway (hsa05417), Fluid shear stress and atherosclerosis (hsa05418), AGE-RAGE signaling pathway in diabetic complications (hsa04933), Prostate cancer (hsa05215), Hepatitis B (hsa05161), Chemical carcinogenesis-receptor activation (hsa05207), Bladder cancer (hsa05219), Human cytomegalovirus infection (hsa05163), Kaposi sarcoma-associated herpesvirus infection (hsa05167), and Hepatitis C (hsa05160) (Fig. 5C and D).

### 3.5. Molecular docking verification

Because the top 10 signaling pathways are directly involved in hepatitis diseases in network pharmacology, we focused on using the key targets in these signaling pathways for molecular docking to verify the combination of the bioactive components in RRH and the gene targets of hepatitis.

**Figure 6.**
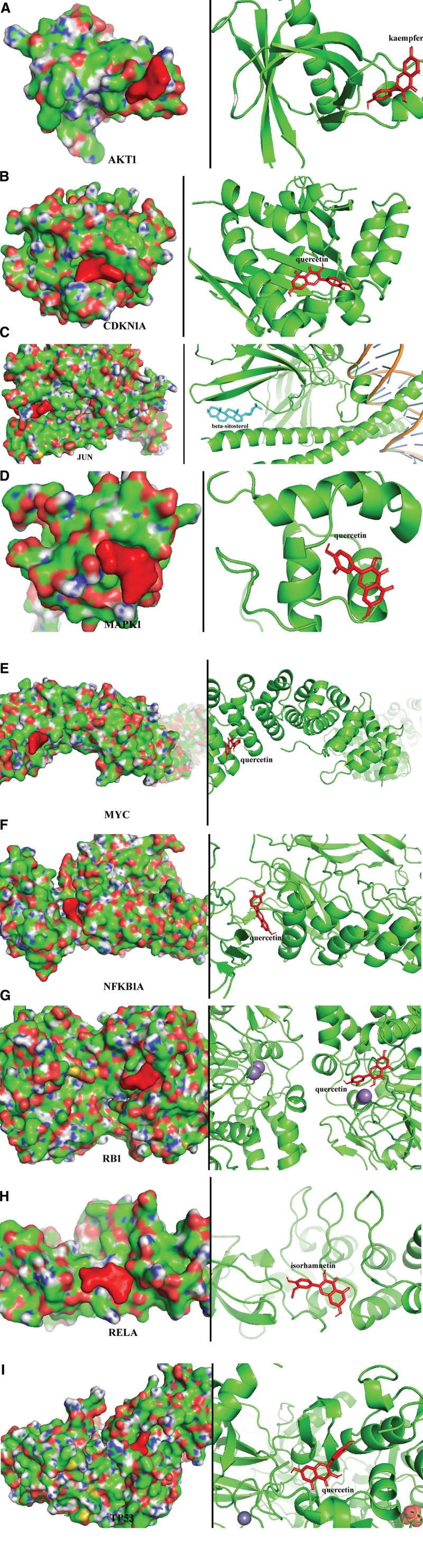
Molecular docking of the hub targets with bioactive components. (A) Binding poses of kaempferol complexed with AKT1, affinity = −6.1 kcal/mol; (B) Binding poses of quercetin complexed with CCND1, affinity = −6.9 kcal/mol; (C) Binding poses of beta-sitosterol complexed with JUN, affinity = −7.1 kcal/mol; (D) Binding poses of quercetin complexed with MAPK1, affinity = −5.5 kcal/mol; (E) Binding poses of quercetin complexed with MYC, affinity = −7.2 kcal/mol; (F) Binding poses of quercetin complexed with NFKBIA, affinity = −7.9 kcal/mol; (G) Binding poses of quercetin complexed with RB1, affinity = −8.0 kcal/mol; (H) Binding poses of isorhamnetin complexed with RELA, affinity = −6.1 kcal/mol; (I) Binding poses of quercetin complexed with TP53, affinity = −7.5 kcal/mol.

Key targets, including JUN, RELA, AKT1, CDKN1A, MAPK1, RB1, TP53, NFKBIA, and MYC, were considered candidate genes for molecular docking. We used the main bioactive components, including quercetin (MOL000098), kaempferol (MOL000422), isorhamnetin (MOL000354), and beta-sitosterol (MOL000358), for binding to the hub targets of the disease. The lowest binding energies of CDKN1A, MAPK1, RB1, TP53, NFKBIA, and MYC for quercetin were −6.9, −5.5, −8.0, −7.5, −7.9, and −7.2 kcal/mol, respectively. Meanwhile, the docking results showed that there were 1, 2, 3, 2, 3, and 2 hydrogen bonds between quercetin and the proteins mentioned above, respectively. The binding energy of AKT1 for kaempferol was −6.1 kcal/mol with 2 hydrogen bonds. JUN for beta-sitosterol was −7.1 kcal/mol with 0 hydrogen bonds, and RELA for isorhamnetin was −6.1 kcal/mol with 4 hydrogen bonds. The affinities of the combinations of bioactive components and hub targets are shown in Table 3, and the molecular docking results are shown in Figure 6.

**Table 3:**
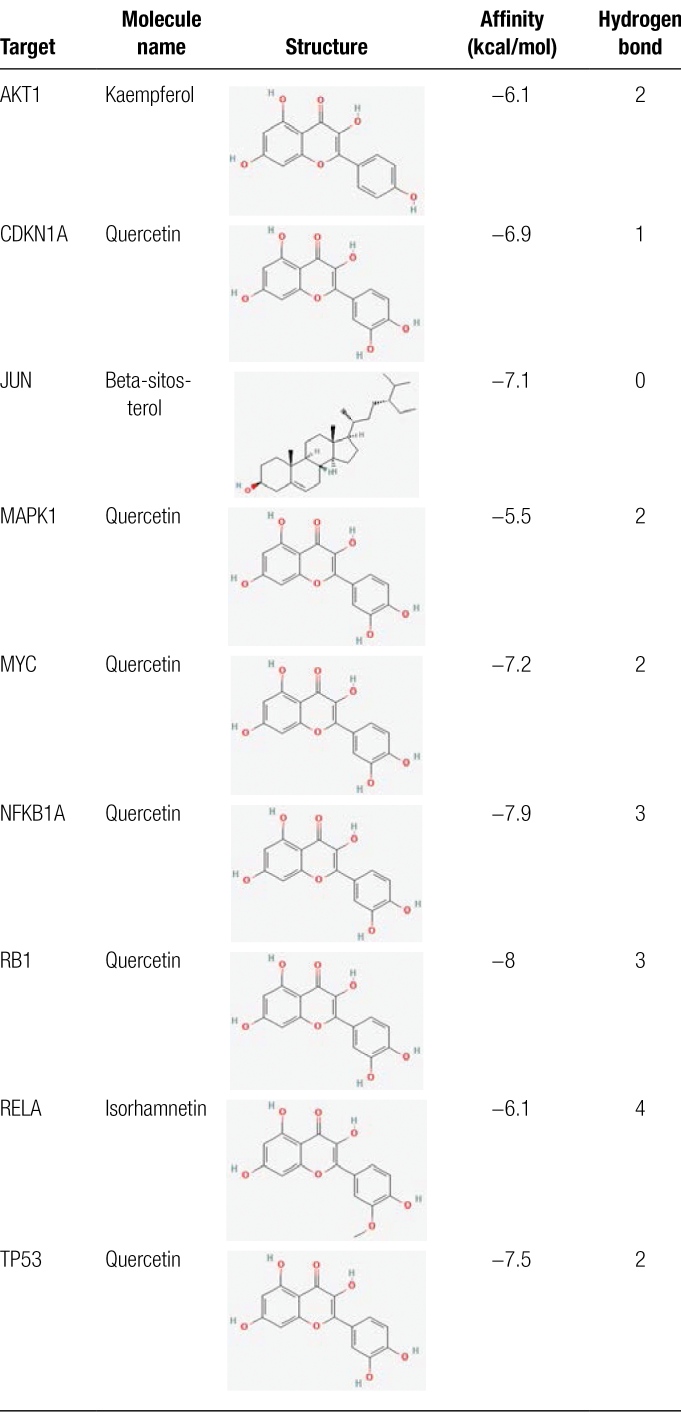
Molecular docking verification between core components and hub targets.

## 4. Discussion

RRH, which is the core medicine of many famous prescriptions including Xiaoyao Powder and Chaihu Shugan Powder, is the classic compatible drug for the treatment of chronic hepatitis. However, the role of RRH in the treatment of chronic hepatitis remains unclear. In this study, we constructed a “components-core gene targets-GO-KEGG” network diagram by screening the core targets of RRH to explore the mechanism of RRH in treating chronic hepatitis. Through a network pharmacological study of RRH in the treatment of chronic hepatitis, we explored the main active ingredients and pharmacological mechanism of RRH, providing a theoretical basis and new targets for follow-up drug research and development.

Topological analysis of the core component network diagram shows the mechanism and function of the core components. According to the degree of composition, the results suggested that quercetin (MOL000098), kaempferol (MOL000422), isorhamnetin (MOL000354), beta-sitosterol (MOL000358), (+)-catechin (MOL000492), 3,5,6,7-tetramethoxy-2-(3,4,5-trimethoxyphenyl) chromone (MOL004598), and petunidin (MOL000490) in RRH may play important roles in the treatment of chronic hepatitis based on our results from network pharmacology. We obtained 14 core targets in the PPI network using the CytoNCA tool in Cytoscape software, including AKT1, MYC, RELA, JUN, MAPK1, FOS, TP53, HIF1A, CCND1, NFKBIA, ESR1, MAPK14, RB1, and CDKN1A. Moreover, the molecular docking results verified the combination of core targets and key ingredients, including quercetin (MOL000098), kaempferol (MOL000422), isorhamnetin (MOL000354), and beta-sitosterol (MOL000358).

By constructing a “components-core targets-GO-KEGG” network diagram, we have visualized the core therapeutic targets of multiple components in RRH, and explored the potential BPs and signaling pathways for the treatment of chronic hepatitis. Through GO enrichment analysis, we found that the top GO BPs, including response to lipopolysaccharide (GO:0032496), response to molecule of bacterial origin (GO:0002237), response to nutrient levels (GO:0031667), response to oxygen levels (GO:0070482), response to hypoxia (GO:0001666), cellular response to chemical stress (GO:0062197), response to decreased oxygen levels (GO:0036293), and response to reactive oxygen species (GO:0000302), may be the main BPs involved in the treatment of chronic hepatitis. At the same time, KEGG enrichment analysis revealed the potential signaling pathways of RRH in the treatment of chronic hepatitis, including Hepatitis B (hsa05161), Hepatitis C (hsa05160), Chemical carcinogenesis-receptor activation (hsa05207), Lipid and atherosclerosis signaling pathway (hsa05417).

Among the classic famous prescriptions for the treatment of chronic hepatitis, Radix Bupleuri plays a major role, and Radix Paeoniae Alba plays an auxiliary role, which was verified by our research results. Among the 7 core components of RRH, Radix Bupleuri contains 4 components, whereas Radix Paeoniae Alba has 2 components, both of which share 1 component. The 2 components of Radix Paeoniae Alba play an auxiliary role in treating hepatitis by binding targets AKT1 and JUN and activating Hepatitis B, Hepatitis C, and other pathways. Furthermore, the 4 components of Radix Bupleuri can combine with most of the core targets to activate most pathways related to the disease and play a vital role in the treatment of hepatitis.

KEGG enrichment analysis revealed that the key components of RRH, including quercetin, kaempferol, isorhamnetin, beta-sitosterol, (+)-catechin, 3,5,6,7-tetramethoxy-2-(3,4,5-trimethoxyphenyl) chromone, and petunidin, could play an anti-hepatic fibrosis role through several key BPs. Quercetin is a natural flavonoid with multiple functions and targets, including antibacterial, anti-inflammatory and immune-regulatory effects. Quercetin has been proven to be used for the treatment of hepatitis B for many years.^[21]^ Recent studies have shown that quercetin has direct and host-mediated antiviral effects on HCV and can also inhibit drug-mediated hepatitis.^[22]^ Recent studies have shown that quercetin, kaempferol and isorhamnetin can interfere with viral Pol/RT and core proteins, thus exerting an anti-HBV therapeutic effect.^[23,24]^ The binding energy of molecular docking is the most direct means to evaluate its outcomes. A lower binding energy corresponds to a stronger binding between the ligand and the receptor. Our molecular docking results demonstrated consistently lower binding energies, indicating stable conformations and strong ligand-receptor interactions. Our molecular docking analysis also showed that quercetin can combine with CDKN1A, MAPK1, RB1, TP53, NFKBIA, and MYC to activate the Hepatitis B and Hepatitis C pathways for anti-hepatitis. Briefly, the minimum binding energies of CDKN1A, MAPK1, RB1, TP53, NFKBIA, and MYC for quercetin were −6.9, −5.5, −8.0, −7.5, −7.9, and −7.2 kcal/mol, respectively. Meanwhile, the docking results showed that there were 1, 2, 3, 2, 3, and 2 hydrogen bonds between quercetin and the proteins mentioned above, respectively, which suggested that quercetin may have a better binding activity to RB1 and NFKBIA. Beta-sitosterol is the main phytosterol found in plants and has extensive protective effects against various chronic diseases. The binding energy of JUN for beta-sitosterol were −7.1 kcal/mol. As an important component of RRH, beta-sitosterol can combine with JUN to play an anti-hepatitis role. Furthermore, beta-sitosterol may play an effective protective role against nonalcoholic fatty liver disease through lipid and atherosclerosis, fluid shear stress, and atherosclerosis signaling pathways.^[25]^ Natural flavonoids (catechins) have been shown to have various pharmacological activities for liver protection. Catechin has potent antioxidant activity and a protective effect on the plasma membrane structure of hepatocytes, thus exerting an inhibitory effect on hepatitis.^[26,27]^ 3,5,6,7-tetramethoxy-2-(3,4,5-trimethoxyphenyl) chromone, which contains high levels of chromones, is known to have anti-inflammatory and anti-hepatoma effects. Moreover, it has been reported that chromone has strong free radical scavenging activity in vitro, which could alleviate hepatic inflammation, inhibit hepatocyte apoptosis, and reduce alcohol-induced histological alteration and lipid accumulation in the liver tissues, thus having an anti-hepatitis effect.^[28,29]^ Our results also confirmed that chromone has a significant anti-hepatitis effect; therefore, 3,5,6,7-tetramethoxy-2-(3,4,5-trimethoxyphenyl) chromone may be a novel agent for hepatitis therapy. Petunidin, a member of the anthocyanin family, is considered to be an effective anti-oxidant. The protective effect of petunidin on liver may be achieved by reducing oxidative stress and inhibiting ROS production, thus treating chronic hepatitis.^[30]^

The formation and progression mechanisms of chronic hepatitis, which are caused by various factors including hepatitis virus, autoimmune factors, and lipid disorders, etc, are complicated.^[31]^ The development of chronic hepatitis is related to a few BPs. The results of GO enrichment analysis showed that the drug plays a role in the treatment of hepatitis in BPs involving response to lipopolysaccharide, response to nutrient levels, response to oxidative stress, and cellular response to chemical stress, etc. In short, RRH may treat hepatitis by inhibiting oxidative stress, reducing chemical damage to the liver, regulating lipid metabolism, and supplying oxygen and nutrition to the liver. KEGG pathway analysis revealed that exerts its effects via multiple signaling pathways, including Hepatitis B, Hepatitis C, Chemical carcinogenesis-receptor activation, lipid and atherosclerosis, and other disease pathways. The results of PPI network topology analysis showed that the therapeutic effect of this drug on hepatitis was mainly achieved by regulating RELA, AKT1, JUN, MAPK1, TP53, CCND1, MYC, RB1, MAPK14, NFKBIA, CDKN1A, HIF1A, ESR1, and FOS. Our results show that RRH has a significant improved liver protection. Network pharmacology research revealed that the effects on liver protection of RRH are characterized by multi-component, multi-target, and multi-path mechanisms of action. The results showed that RRH screened a total of 19 active ingredients and 176 targets, participated in inhibiting oxidative stress, reducing chemical damage to the liver, regulating lipid metabolism, and supplying oxygen and nutrition to the liver and other BPs, and mainly involved multiple signaling pathways to jointly exert liver protection effects.

Molecular modeling proves to be an efficient method for screening potential new compounds in the search for anti-hepatitis capabilities.^[32]^ By screening the TCMSP database, we were able to identify various pharmacological components with anti-hepatitis activity. Subsequently, we conducted further analysis on the binding mode of core compounds using molecular docking. Research has shown that piperine antimicrobial activity and cytotoxicity effects can be verified through molecular modeling.^[33,34]^ Additionally, molecular modeling can also confirm the clinical therapeutic effects of different components such as geissoschizoline N4-methylchlorine and Eleutherine plicata by simulating molecular docking.^[35,36]^ Consequently, in silico modeling offers the potential to develop new drug candidate ingredients with targeted effects.^[37]^

Although network pharmacology offers a visual network diagram of the mechanism of RRH in the treatment of chronic hepatitis, it still has some limitations. First, the authenticity and reliability of network pharmacology need to be verified by animals and experiments because network pharmacology is based on database analysis. Second, the active ingredients of the drug are affected by the dose, and the key ingredients shown in network pharmacology may not play an important role in clinical practice. To solve these problems, network pharmacology results of chronic hepatitis need be carried out to verify the mechanism of action of effective ingredients on chronic hepatitis and provide a theoretical basis for new drug research and development.

## 5. Conclusions

In conclusion, our results revealed that RRH in the treatment of hepatitis was characterized by multi-component, multi-target, and multi-path mechanisms of action. Although our results are based on database analysis and lack the influence of drug dosage, network visualization shows the anti-hepatitis effect of key components. Furthermore, verification is needed through animal experiments in the future. The main bioactive components in RRH, including quercetin, kaempferol, isorhamnetin, and beta-sitosterol, were used to bind the hub targets of the disease, which may provide a theoretical basis for the development of multi-component, multi-target, and multi-path drugs for hepatitis.

## Author contributions

**Conceptualization:** Long Huang.

**Data curation:** Long Huang, Qingsheng Yu, Zhou Zhen.

**Formal analysis:** Long Huang.

**Funding acquisition:** Long Huang, Qingsheng Yu.

**Methodology:** Hui Peng.

**Project administration:** Long Huang, Qingsheng Yu, Zhou Zhen.

**Resources:** Long Huang, Hui Peng, Zhou Zhen.

**Software:** Long Huang, Hui Peng, Zhou Zhen.

**Supervision:** Qingsheng Yu.

**Validation:** Long Huang, Qingsheng Yu, Zhou Zhen.

**Visualization:** Long Huang, Hui Peng.

**Writing – original draft:** Long Huang.

**Writing – review & editing:** Qingsheng Yu, Hui Peng, Zhou Zhen.
